# Management of Pelvic Fracture Urethral Injury: Is Supracrural Urethral Rerouting (Step 4) Becoming Anecdotical or Does It Remain in Force?

**DOI:** 10.3390/jcm12062427

**Published:** 2023-03-22

**Authors:** Christian Yepes, Maciej Oszczudlowski, Marco Bandini, Pankaj M. Joshi, Ahmed Alrefaey, Shreyas Bhadranavar, Francisco E. Martins, Sanjay B. Kulkarni

**Affiliations:** 1 Department of Urology, Kulkarni Reconstructive Urology Center, Pune 411045, India; 2 Urology Clinic, Centre of Postgraduate Medical Education, 01-813 Warsaw, Poland; 3 Unit of Urology, Urological Research Institute (URI), Vita-Salute San Raffaele University, San Raffaele Hospital, 20132 Milano, Italy; 4 Department of Urology, School of Medicine, University of Lisbon, Hospital Santa Maria, 1649-028 Lisboa, Portugal

**Keywords:** posterior urethra, pelvic fracture urethral injury, perineal approach, transpubic approach, anastomotic urethroplasty, supracrural urethral rerouting

## Abstract

Webster described a step-based perineal approach for repairing the posterior urethra in patients with pelvic fracture urethral injury (PFUI). The higher the complexity of the step, the higher the morbidity for the patient and the lower the surgical outcomes. We evaluated the outcomes of anastomotic urethroplasty (especially Step 4 or higher) or substitution urethroplasty in patients with PFUI at our center. Between 2013 to 2021, we retrospectively collected data on patients with PFUI. Surgical procedures were categorized according to the Webster classification and rates of each step were reported. The success rate was defined as Qmax above 10 mL/s and no need for further treatment. In this period, 737 male patients with PFUI were surgically treated. Notably, 18.8%, 17.6%, 46%, 1.8%, and 5.6% of included patients received steps 1, 2, 3, and 4 and the abdominoperineal approach, respectively. In 68 (9.2%) patients, the substitution of urethroplasty with a pedicled preputial tube (PPT) was needed. The success rate was 69.2% in Step 4, 74.4% in the abdominoperineal approach, and 86.4% in PPT; however, recurrence-free survival was not significantly different between groups (*p* = 0.22). Step 4 perineal anastomotic urethroplasty represents a surgical option in the armamentarium of PFUI treatment. Indications should be carefully reviewed to improve patient selection and avoid surgical failure, stopping at the step which first gives a tension-free anastomosis.

## 1. Introduction

The incidence of posterior urethral injuries after pelvic fracture ranges from 3% to 25%, depending on the study and the specific type of fracture [1]. It is one of the most debilitating situations in urology and represents a real surgical and decision-making challenge [2]. The site of urethral injury is the bulbo-membranous junction in most patients [3,4]. Rarely, the urethral injury is localized in the prostate-membranous junction, as can happen in children [5], intraprostatic, and/or at the bladder neck.

In 1986, George D. Webster described a one-stage perineal end-to-end anastomotic repair of urethral stenosis [6]. Later on, the same author developed an elaborated perineal approach that comprises four surgical steps that are used sequentially, as required, depending on the magnitude of the urethral defect to accomplish a tension-free bulbo-prostatic urethral anastomosis [7]. These steps are circumferential urethral mobilization (Step1), corporeal body separation (Step 2), inferior wedge pubectomy (Step 3), and supracrural urethral rerouting (Step 4). Nevertheless, an abdominoperineal approach can be still necessary to address previous failed primary repairs, intraprostatic urethral ruptures, or recto-urethral fistulae. These steps are characterized by higher surgical complexity and they carry greater patient morbidity [8]. Given these premises, Step 4 (supracrural urethral rerouting to the anastomosis) or the abdominoperineal approach with or without an omental wrap is performed only in selected patients where no other option is possible [2].

The aim of this study is to evaluate outcomes of anastomotic urethroplasty after PFUI with Step 4 and higher or substitution urethroplasty with a pedicled preputial tube and to assess the utility of Step 4 in a contemporary cohort of patients.

## 2. Materials and Methods

### 2.1. Study Population

This is a retrospective observational study including data from a prospectively maintained urethroplasty database of 737 male patients with PFUI treated at our tertiary care reconstructive center between 2013 to 2021. Case sheets and other patient-related documents were reviewed. Patients with PFUI secondary to blunt pelvic trauma and complete clinical records available were included in the study. 

### 2.2. Preoperative Evaluation

Patients were assessed with clinical history, physical examination, urinalysis, urine culture, uroflowmetry, postvoid residual urine measurement (ultrasound), and penile doppler in adults (age ≥ 18 years) to document flow in the dorsal and cavernosal penile arteries. Assessment of stricture length was carried out using retrograde urethrography (RGU) and voiding cystourethrography (VCUG) on the day of surgery. Moreover, urethrocystoscopy with the use of small caliber rigid/flexible endoscopes to evaluate stricture length, caliber, and the appearance of the urethral mucosa and the bladder neck was performed in each patient prior to the surgery [9]. Abdominal and pelvic computed tomography (CT) and magnetic resonance urethrography (MRU) were completed in selected cases [10].

### 2.3. Clinical Outcomes 

Surgical procedures for anastomotic urethroplasty were categorized according to the Webster classification (elaborated perineal approach) [6,11]: Step 1: urethral mobilization; Step 2: corporal body separation; Step 3: inferior wedge pubectomy with substeps (3a periosteal elevation, 3b-inferior wedge pubectomy); Step 4: supracrural urethral rerouting and combined abdominoperineal approach with or without an omental wrap. Rates of each step were compared. We analyzed the baseline demographic parameters (age, stenosis etiology, length of defect, site of injury, and previous interventions performed), treatment details (surgical approach and ancillary maneuvers), and postoperative complications. 

Postoperative follow up included office visits after hospital discharge with the first visit on day 7. The urethral catheter was kept for 4 weeks in anastomotic urethroplasty, and 6 weeks following substitution urethroplasty (PPT). During urethral catheter removal, patients performed a uroflowmetry and if the flow was considered satisfactory (maximum urinary flow rate [Qmax] ≥10 mL/s or rating 1 or 2 according to the Urethral Stricture Surgery Patient-Reported Outcome Measures [USS-PROM] questionnaire scale in question 8) [12], the suprapubic catheter (SPC) was usually removed at the same time. Urethrogram was performed only when Qmax was below 10 mL/s and in case of suspected fistulae or failure. 

After catheter removal, patients were scheduled for follow-ups at 3, 6, and 12 months, and yearly thereafter. Patients were followed-up at our center, when practical and feasible, or with the help of the referring urologist. To overcome the challenges from travel constraints, we preferred internet-based applications such as e-mail and WhatsApp™, to acquire follow-up uroflowmetry and postoperative symptoms data. This has been significant progress in achieving patient follow up. 

Physical exam, uroflowmetry, and urinalysis were performed on each visit. If the patient presented low urinary tract symptoms (LUTS), an abnormal uroflowmetry (peak flow <10 mL/s), obstructive voiding curve, or recurrent urinary tract infection (UTI) a diagnostic RGU/VCUG was requested, followed by urethrocystoscopy if needed. 

We utilized the USS-PROM form and erectile dysfunction (e.g., International Index of Erectile Function [IIEF-5]) [13] after surgical reconstruction on e-mail or WhatsApp™ messages for achieving follow up. 

The success rate was defined as peak flow (Qmax) above 10 mL/s and no need for further urethral instrumentation post-operatively. 

Lost to follow up was defined as when a patient did not report his status 2 years after the last consultation.

### 2.4. Statistical Tests 

Descriptive statistical analysis was made. Medians and interquartile ranges (IQRs) are reported for continuously coded variables. Time-to-event analyses were conducted using a Kaplan–Meier analysis with log-rank test. The level of statistical significance was set at *p* < 0.05. For all statistical analysis, R program with survminer package was used.

## 3. Results

### 3.1. Baseline Characteristics

A total of 737 male patients with a minimal follow up of 6 months underwent anastomotic urethroplasty due to PFUI from January 2013 to October 2021. The median age of presentation in our series was 28 years (range 7–72), median defect lengths were 3 cm (range 1–6) and 6 cm (range 1–12) in primary and redo cases, respectively, and median follow up was 48 months (range 6–108). Stricture location was posterior urethra in all cases and all patients had a history of prior pelvic blunt trauma. The baseline patient characteristics are depicted in Table 1. 

### 3.2. Urethroplasty Outcomes 

**Figure 1:**
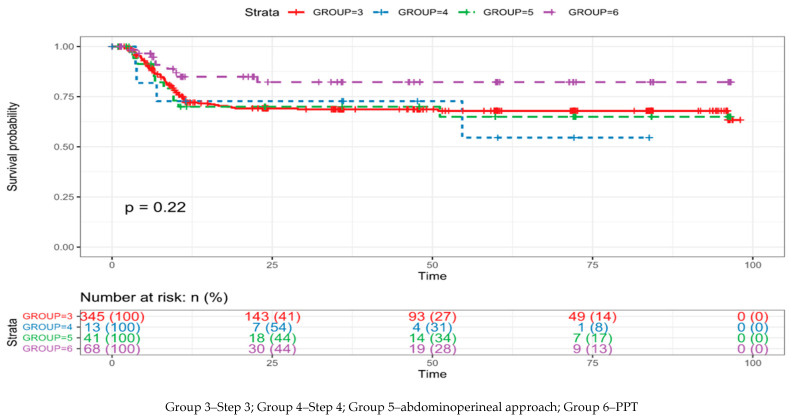
Kaplan–Meier curves for anastomotic urethroplasty with steps 3 and higher and for PPT. Time in months.

**Figure 2:**
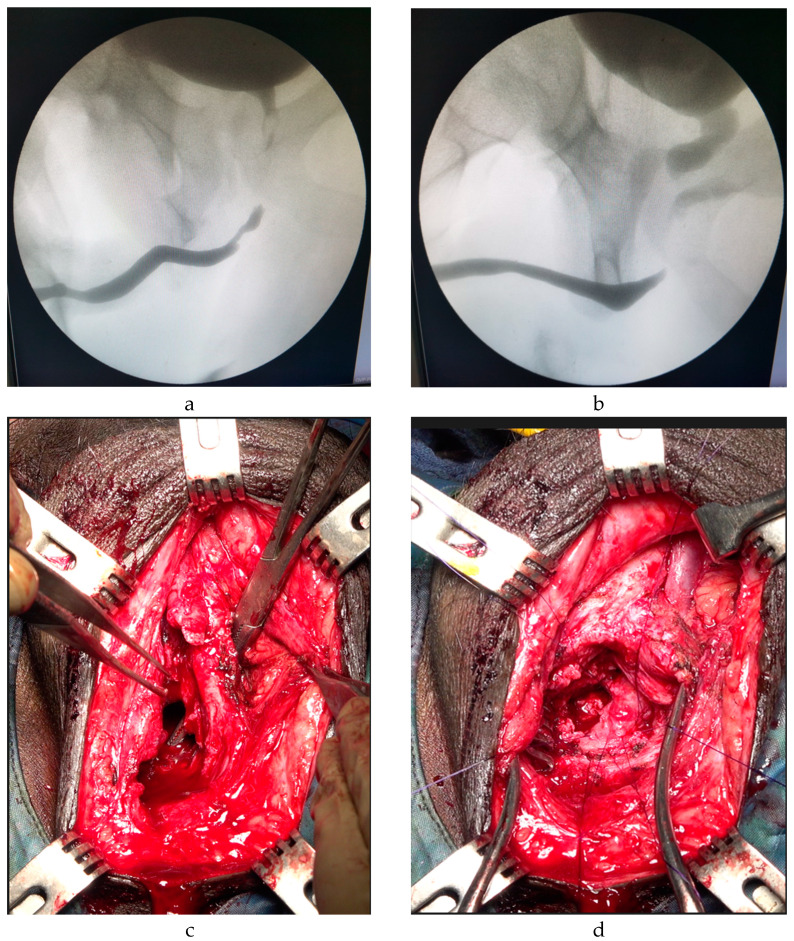
RGU/VCUG presenting normal anterior urethra, long gap, and bladder neck open (**a,b**). The urethra is routed around the lateral side of the left corporal body at the peno- bulbar junction to straighten the natural curve of the bulbar urethra completely reducing further the distance to the anastomosis. Then through the bony defect created by the earlier inferior pubectomy. A small furrow of the bone should be gouged from the pubis where the urethra runs, to avoid its compression between the corpus and bone (**c**,**d**).

577 (78.2%) of 737 patients were successful. Among those, 140 (18.8%) received Step 1, 130 (17.6%) received Step 2, 345 (46.8%) received Step 3a (34) or 3b (311), 13 (1.8%) received Step 4, and in 41 (5.6%) urethroplasty utilizing combined abdominoperineal approach with or without an omental wrap. In this time span, the number of patients receiving substitution urethroplasty with PPT was 68 (9.2%) when the bulbar urethra was necrotic. Success rates in each group are presented in Table 2. Of all patients, 147 (19.9%) were defined as lost at follow up. Kaplan–Meier curves for anastomotic posterior urethroplasty with steps 3 and higher and for PPT are presented in Figure 1. Median survival time could not be calculated because more than half of the patients were still free of recurrence (successful) when censored. Differences between the last-mentioned groups were not statistically significant in terms of recurrence-free survival (*p* = 0.22). The surgical failure occurred in most patients within the first year after anastomotic urethroplasty (Figure 2) and usually manifested as an anastomotic ring (data are not presented).

Among patients who received Step 4 anastomotic urethroplasty (Figure 2, Table 3), the median age was 19 years (range 9–40). Eleven (84.6%) patients underwent one or multiple attempts of PFUI repair before definitive surgery, with a success rate of 53.8%. Only 2 (15.4%) patients were primary cases, with a success rate of 100% after repair. A validated psychometric questionnaire (IIEF-5) was completed by sexually active patients (all patients in the Step 4 group). Notably, 6 (46.1%) of them had preoperative mild to moderate erectile dysfunction. After surgery, one patient had severe erectile dysfunction, needing subsequent placement of a malleable penile prosthesis.

All patients in whom urethroplasty with abdominoperineal approach was performed, presented complex PFUI such as multiple failed prior urethroplasty (re-redo), ischemic narrowing or necrosis of the bulbar urethra, double block at the bulbo-membranous junction and bladder neck–prostate, recto-urethral fistula, incontinence due to bladder neck injury and patients who have a concomitant posterior urethral injury with anterior urethral strictures.

A successful posterior urethral repair rate was achieved in 9 of the 13 patients (69.2%) in Step 4 and in 30 of the 41 patients (74.4%) with an abdominoperineal approach. Stenosis recurrences were observed in 30.8% (4) and 25.6% (11) in Step 4 and abdominoperineal approach, respectively, defined as abnormal Qmax or/and the need for further urethral instrumentation (Table 2).

Our algorithm is to treat these patients with one endoscopic direct visual internal urethrotomy (DVIU). If the patient recurred after DVIU, a redo urethroplasty is undertaken.

Among failed patients with supracrural urethral rerouting, 4 (100%) patients underwent redo anastomotic urethroplasty, which was successful for 2 (50%) of them, for a final success rate of 84.6%. In both cases, direct anastomosis was achieved with no further ancillary maneuvers.

The success rate after Step 3 of anastomotic urethroplasty or inferior was higher compared to Step 4, abdominoperineal approach, and pedicled preputial tube, 79.8% (479) vs. 76.6% (98), respectively.

## 4. Discussion

The etiology of pelvic fracture-related injuries to the urinary tract is, in the majority, road traffic accidents. In developing nations, cases are more often related to work and traffic accidents (pedestrians or on two-wheel transportation), falls from heights, or natural catastrophes [14]. Many of these injuries are complex in nature, including concomitant rectal perforation, bladder neck disruption, fistula, or abscess [15]. Management of posterior urethral disruption defects is quite challenging, and different techniques have been proposed with variable long-term results [16]. The basis for the progressive elaborated perineal approach described by Webster is to alter the geometry of the path needed to approximate the bulbar urethral end to the dislocated proximal urethral stump [8]. This stepwise anastomotic urethroplasty remains the gold standard for the treatment of PFUI.

Koraitim et al. reported using clinico-radiological variables, including a gapometry/urethrometry (G/U) index, urethral gap length, and prostate displacement, to predict which patients might be manageable by a simple perineal anastomotic urethroplasty alone (index < 0.35) and which would require an elaborated perineal or transpubic approach (index > 0.35) [17]. The G/U index has the highest predictive accuracy for surgical repair, with 91% specificity, 90% sensitivity, and 95% positive predictive value. We do not use the G/U index routinely to predict the surgical approach. We believe that even for the most experienced reconstructive surgeon, conventional preoperative imaging can give an indication, but no certainty, of the surgical approach. Different factors (trauma kinematics, complexity of PFUI, and patient anthropometry) have a direct influence on the choice of surgical approach. The main factor is the experience of the surgeon to evaluate the grade of tension of the anastomosis (completely subjective) and the familiarity with the ancillary maneuvers to decide the correct approach.

In 2003, an update of the experience of Webster, Flynn et al. reported the long-term result of this progressive one-stage perineal anastomosis repair in 120 patients with PFUI, with a success rate of 92.5% [18]. They noted a chronological progression towards more elaborated repairs to achieve tension-free anastomosis due to a change in demographics and increased confidence in the safety and success of the progressive perineal approach (16). Reports from different reconstructive centers have challenged the surgical practicability of supracrural urethral rerouting, reporting that this step was rarely recommended, and successful outcome was low (17–20). Kizer et al. in a retrospective study of 142 males with PFUI treated with anastomotic urethroplasty, performed Step 1 in 95 patients (67%), Step 2 in 24 (17%), Step 3 in 14 (10%), Step 4 in 4 (3%) and abdominoperineal approach in 5 (4%). Notably, 3/4 patients (75%) who received Step 4 experienced recurrent stenosis. They concluded that urethral rerouting appears to be inferior to the abdominoperineal approach as a salvage maneuver for complex cases, and did not appear to enhance effectiveness (25% success rate) [19].

In 2009 Hosseini et al. in a retrospective review of 200 men treated with anastomotic urethroplasty for PFUI, Step 1 was performed in 79 (39.5%), Step 2 in 69 (34.5%), Step 3 in 22 (11.0%), Step 4 only in 11 (5.5%) of whom 7 (63.6%) sustained recurrent stenosis requiring intervention, and a combined abdominoperineal procedure in 19 (9.5%) which was successful in 15 (78.9%). The authors concluded that Step 4 is not an acceptable technique (success rate 18.2%) and can result in significant postoperative complications [20].

In a retrospective study in 2010, Singh et al. reported their experience in 172 patients undergoing perineal urethroplasty for posterior urethral injuries. They found corporal rerouting needed in 3 patients (1.7%) with a success rate of 66.7% [21]. Fu et al. reported a series of 301 patients, with 263 (87.4%) successes. Step 1 was used in 103 (34.2%), Step 2 in 89 (29.6%), Step 3 in 95 (31.6%), and Step 4 in 14 (4.7%). Success rates were 89.3%, 86.5%, 84.2%, and 85.7% for steps 1, 2, 3, and 4, respectively [21].

In the opposite direction in 2015, Webster presented a review of its original 4-step progressive elaborated approach to repair of PFUI, asserting that despite recently in developed countries there has been a move to minimize the steps required to achieve a tension-free anastomosis, primarily by avoiding supracrural rerouting of the urethra, it would be a mistake to disregard this technique [8].

For the management of PFUI, we support the use of each step on the principle of accomplishing adequate proximal urethral exposure for correct scar excision and guaranteeing tension-free anastomosis instead of providing additional urethral length. In our hands, Step 4 anastomotic urethroplasty was rarely required (1.8%) (Table 4). The reasons behind this decline are several, including the improved technique of inferior pubectomy. Moreover, distortion of normal urethral anatomy after Step 4 is significantly altered, making any surgical revision, and possibly any attempt to perform lower urinary tract instrumentation if needed posteriorly, extremely challenging. Furthermore, there are several possible complications related to the procedure such as torsion, ischemic strictures of the rerouted bulbar urethra, or injury of the cavernous nerves. It also showed lower success rates compared to steps 1, 2, and 3. However, Step 4 avoids the abdominal approach and superior pubectomy, which are usually associated with higher morbidity for the patient.

We do not take a position in favor or against Step 4 anastomotic urethroplasty since it clearly represents a surgical option in the armamentarium of PFUI treatment, even though it is less needed. In practical terms, when the gap is long, it can be bridged without tension by means of an inferior pubectomy. In most cases, if this is not possible, the majority will require the transpubic approach, which is clearly highlighted by the data presented in the study. On the other hand, indications for Step 4 should be carefully reviewed to improve patient selection and avoid surgical failure.

First, the blood supply to the bulbar urethra should be intact. Multi-stage surgery or Step 4 is contraindicated for patients with bulbar urethral necrosis, where, instead, single-stage substitution urethroplasty with pedicled flaps from prepuce or distal penile skin is recommended [23]. To this aim, we firmly recommend evaluating all adult patients with preoperative color doppler of the cavernosal and dorsal penile arteries before and after administration of intracavernosal Prostaglandin E1, which provides information on the vascularity of the bulbar urethra, irrespective of the status of erectile function. This assessment can guide surgeons during their dissection of the posterior urethral stump, preserving the side where the dorsal penile artery showed better flow and deepening the dissection on the side with the decreased flow. Moreover, examination of the vascular supply with the penile doppler ultrasound [24] may reduce the risk of postoperative erectile dysfunction (ED), since the etiology of ED in patients with PFUI can be vasculogenic or neurogenic secondary to the initial trauma, or iatrogenic secondary to injury of penile vessels during dissection of the posterior urethra.

Second, the length of the bulbar urethra should be carefully assessed during surgery. The bulbar urethra should be defined as the segment beginning in the penoscrotal junction and ending at the bulbo-membranous junction, instead of only the segment that lies within the bulbospongiosum muscle. This means that the longer the penile length, the longer the bulbar urethral length and the more elastic it is [25]. A tension-free anastomosis is pivotal, especially in Step 4, where tension is typically associated with anastomosis disruption and failure. Indeed, in Step 4, the urethra runs around the corpora, thus erections more easily cause compression and stretching of the urethra, facilitating anastomosis ruptures and failure [25].

Third, Step 4 avoids the transabdominal approach; thus, its use can be favored in patients with no injury to the prostatic urethra or bladder neck where the transabdominal approach will only have the purpose of reapproximating the proximal urethra stump without exposing it to significant morbidity and higher postoperative complications.

Fourth, if tension-free anastomosis can be reached with Step 4, a pedicled preputial tube can be avoided. This has the great advantage of preventing PPT-related complications, such as recurrent UTI, diverticulum formation, and stone formation (Table S1). Post-micturition dribbling is not considered a complication, instead is a common self-limiting side effect managed with the manual evacuation of urine. In our study group, these complications were Grade II and III according to the Clavien-Dindo classification [26] and were managed conservatively and surgically. UTI was managed with antibiotics, diverticulum with diverticulectomy, and urethral stones with endoscopic laser lithotripsy.

There are opposite positions in the literature regarding predictors of surgical approach for PFUI repair. Andrich et al. concluded that it is not possible to predict in advance the surgical procedure needed to achieve a tension-free anastomosis based on the measured length of the radiological defect, except at the extremes of length (<1 cm or >4 cm). Unpredictability is due to different factors such as fibrosis, loss of urethral length, displacement of the bladder and prostate, and the elasticity of the dorsal urethra [27].

Koraitim found three independent predictive factors of the surgical approach: G/U index, urethral gap length, and prostatic displacement. Concluding that G/U was the most accurate predictor [17]. Recently, Johnsen et al., in a retrospective multi-institutional study, found that patients requiring ancillary maneuvers tended to be younger, have longer distraction defects, and have a history of angioembolization. Patients with angioembolization and increased distraction defect length had more risk of urethroplasty failure [22].

Tension at the anastomosis is obviously a subjective impression except at extremes and it may sometimes be difficult to judge whether a degree of tension is acceptable or whether one should progress to the next tension-relieving step. Additionally, clear familiarity with the next step makes one more inclined to proceed with it, perhaps more inclined than might be necessary. In concordance with Andrich et al. [27], we suggest that the reconstructive surgeon repairing a PFUI must be willing and able to perform all the steps described, preferring one or the other step according to patients' characteristics rather than surgical practicability.

Our study is limited, as it is a retrospective single-center experience study. Most of the patients included were referred to our tertiary high-volume center due to the complexity of the cases, so details of initial trauma severity, stenosis length, previous investigations, and treatments were limited. Following up was challenging due to the fact that the patients are from different geographical areas.

## 5. Conclusions

Step 4 (supracrural urethral rerouting) perineal anastomotic urethroplasty was rarely performed due to the improved technique of inferior pubectomy. Nevertheless, it would be a mistake to disregard Step 4 anastomotic urethroplasty in selected patients as it avoids the transabdominal approach, superior pubectomy, and pedicled preputial tube-related complications and morbidity. Indications should be carefully reviewed to improve patient selection and avoid surgical failure. However, ancillary maneuvers are frequently required, and Step 4 cannot be predicted preoperatively with conventional imagining.

## Figures and Tables

**Table 1:** Cohort characteristics.

Variable	Overall
Urethroplasty	
Primary, n (%)	346 (47)
Redo, n (%)	391 (53)
Age, years, median (IQR)	28 (7–72)
Stricture etiology, n (%)	
Pelvic blunt trauma	737 (100)
Defect size, cm, median (IQR)	
Primary	3 (1–6)
Redo	6 (1–12)
Outcome	
Success, n (%)	577 (78.2)
Recurrence, n (%)	160 (21.8)
Site of injury, n (%)	
Posterior urethra	737 (100)
Initial Management, n (%)	
Suprapubic catheter	700 (95)
Realignment	37 (5)

**Table 2:** Success rate according to surgical technique.

Surgical Procedure	No. of Patients	Success Rate
Circumferential urethral mobilization	140 (18.8%)	104 (74.2%)
Corporeal body separation	130 (17.6%)	118 (91%)
Inferior wedge pubectomy	345 (46.8%)	257 (74.4%)
Substeps	34 (3a), 311 (3b)	
Supracrural urethral rerouting	13 (1.8%)	9 (69.2%)
Abdominoperineal approach	41 (5.6%)	30 (74.4%)
Pedicled preputial tube	68 (9.2%)	59 (86.4%)

**Table 3:** Supracrural urethral rerouting cohort characteristics (N = 13).

Variable	Overall
Median age (range)	19 (9–40)
Stenosis etiology Pelvic trauma	13 (100%)
Primary cases Redo cases	2 (15.4%) 11 (84.6%)
Erectile dysfunction	6 (46.1%)
Success rate Recurrence Final success rate	9 (69.2%) 4 (30.8%) 11 (84.6%)

**Table 4:** Studies on PFUI.

Authors and Year of Publication	Patients (n)	Supracrural Urethral Rerouting (Step 4) (n)	Success Rate (%)
Webster et al. [6], 1991	74	11	87
Flynn et al. [18], 2003	120	46	-
Kizer et al. [19], 2007	142	4	25
Hosseini et al. [20], 2009	200	11	18.2
Singh et al. [21], 2010	172	3	66.6
Kulkarni et al. [14], 2010	255	4	100
Johnsen et al. [22], 2019	122	2	100
Joshi et al. [23], 2023	737	13	84.6

## Supplementary Materials

The following supporting information can be downloaded at: https://www.mdpi.com/article/10.3390/jcm12062427/s1, Table S1: Complications of PPT according to Clavien-Dindo classification.

## References

[1] Latini J.M., McAninch J.W., Brandes S.B., Chung J.Y., Rosenstein D. (2014). SIU/ICUD Consultation On Urethral Strictures: Epidemiology, etiology, anatomy, and nomenclature of urethral stenoses, strictures, and pelvic fracture urethral disruption injuries. Urology.

[2] Kulkarni S.B., Joshi P., Ramírez Pérez E.A., Martins F.E., Kulkarni S.B., Köhler T.S. (2020). Surgical Reconstruction of Pelvic Fracture Urethral Injury. Textbook of Male Genitourethral Reconstruction.

[3] Andrich D.E., Mundy A.R. (2001). The nature of urethral injury in cases of pelvic fracture urethral trauma. J. Urol..

[4] Mouraviev V.B., Santucci R.A. (2005). Cadaveric anatomy of pelvic fracture urethral distraction injury: Most injuries are distal to the external urinary sphincter. J. Urol..

[5] Sreeranga Y.L., Joshi P.M., Bandini M., Kulkarni S.B. (2022). Comprehensive analysis of paediatric pelvic fracture urethral injury: A reconstructive centre experience. BJU Int..

[6] Webster G.D., Ramon J. (1991). Repair of pelvic fracture posterior urethral defects using an elaborated perineal approach: Experience with 74 cases. J. Urol..

[7] Webster G.D., Goldwasser B. (1986). Perineal transpubic repair: A technique for treating post-traumatic prostatomembranous urethral strictures. J. Urol..

[8] Webster G.D., Peterson A.C. (2015). Simple perineal and elaborated perineal posterior urethroplasty. Arab. J. Urol..

[9] Kulkarni S.B., Bandini M., Patil A., Bhadranavar S., Sharma V., Bafna S., Yatam S.L., Barbagli G., Montorsi F., Joshi P.M. (2021). The Right Instrument for the Right Purpose: Spreading the Use of Small Caliber Ureteroscope for the Inspection of the Male and Female Urethra. Société Int. D’urologie J..

[10] Joshi P.M., Desai D.J., Shah D., Joshi D.P., Kulkarni S.B. (2021). Magnetic resonance imaging procedure for pelvic fracture urethral injuries and recto urethral fistulas: A simplified protocol. Turk. J. Urol..

[11] Joshi P., Bandini M., Montorsi F., Kulkarni S.B. (2022). Challenging the dogma of 6 steps for anastomotic urethroplasty in posterior urethral stricture: Introducing step 3a. World J. Urol..

[12] Jackson M.J., Sciberras J., Mangera A., Brett A., Watkin N., N'Dow J.M., Chapple C.R., Andrich D.E., Pickard R.S., Mundy A.R. (2011). Defining a patient-reported outcome measure for urethral stricture surgery. Eur. Urol..

[13] Rosen R.C., Riley A., Wagner G., Osterloh I.H., Kirkpatrick J., Mishra A. (1997). The international index of erectile function (IIEF): A multidimensional scale for assessment of erectile dysfunction. Urology.

[14] Kulkarni S.B., Barbagli G., Kulkarni J.S., Romano G., Lazzeri M. (2010). Posterior urethral stricture after pelvic fracture urethral distraction defects in developing and developed countries, and choice of surgical technique. J. Urol..

[15] Barbagli G. (2007). History and evolution of transpubic urethroplasty: A lesson for young urologists in training. Eur. Urol..

[16] Cooperberg M.R., McAninch J.W., Alsikafi N.F., Elliott S.P. (2007). Urethral reconstruction for traumatic posterior urethral disruption: Outcomes of a 25-year experience. J. Urol..

[17] Koraitim M.M. (2009). Predictors of surgical approach to repair pelvic fracture urethral distraction defects. J. Urol..

[18] Flynn B.J., Delvecchio F.C., Webster G.D. (2003). Perineal repair of pelvic fracture urethral distraction defects: Experience in 120 patients during the last 10 years. J. Urol..

[19] Kizer W.S., Armenakas N.A., Brandes S.B., Cavalcanti A.G., Santucci R.A., Morey A.F. (2007). Simplified reconstruction of posterior urethral disruption defects: Limited role of supracrural rerouting. J. Urol..

[20] Hosseini S.J., Rezaei A., Mohammadhosseini M., Rezaei I., Javanmard B. (2009). Supracrural rerouting as a technique for resolution of posterior urethral disruption defects. Urol. J..

[21] Singh S.K., Pawar D.S., Khandelwal A.K., Jagmohan (2010). Transperineal bulboprostatic anastomotic repair of pelvic fracture urethral distraction defect and role of ancillary maneuver: A retrospective study in 172 patients. Urol. Ann..

[22] Johnsen N.V., Moses R.A., Elliott S.P., Vanni A.J., Baradaran N., Greear G., Smith T.G., Granieri M.A., Alsikafi N.F., Erickson B.A. (2020). Multicenter analysis of posterior urethroplasty complexity and outcomes following pelvic fracture urethral injury. World J. Urol..

[23] Joshi P.M., Bandini M., Yepes C., Bafna S., Bhadranavar S., Sharma V., Cirulli G.O., Kulkarni S.B. (2022). Flaps for bulbar urethral ischemic necrosis in pelvic fracture urethral injury. Plast. Aesthetic Res..

[24] Guan Y., Wendong S., Zhao S., Liu T., Liu Y., Zhang X., Yuan M. (2015). The vascular and neurogenic factors associated with erectile dysfunction in patients after pelvic fractures. Int. Braz. J. Urol..

[25] Joshi P.M., Batra V., Kulkarni S.B. (2019). Controversies in the management of pelvic fracture urethral distraction defects. Turk. J. Urol..

[26] Clavien P.A., Barkun J., de Oliveira M.L., Vauthey J.N., Dindo D., Schulick R.D., de Santibañes E., Pekolj J., Slankamenac K., Bassi C. (2009). The Clavien-Dindo classification of surgical complications: Five-year experience. Ann. Surg..

[27] Andrich D.E., O’Malley K.J., Summerton D.J., Greenwell T.J., Mundy A.R. (2003). The type of urethroplasty for a pelvic fracture urethral distraction defect cannot be predicted preoperatively. J. Urol..

