# Supplementary material for: Management of Pelvic Fracture Urethral Injury: Is Supracrural Urethral Rerouting (Step 4) Becoming Anecdotical or Does It Remain in Force?
Source: J Clin Med. 2023 Mar 22;12(6):2427. doi: 10.3390/jcm12062427 (PMC10058563; doi:10.3390/jcm12062427)
Supplement: Supplementary file 1 [file jcm-12-02427-s001.zip › jcm-2258732-supplementary.pdf]

Table S1. Complications of PPT according to Clavien-Dindo classification.

Complications	Grade II	Cases, n	Grade III	Cases, n
Early	UTI	9/68 (13.2%)		
Late			Diverticulum from PPT	21/68 (30.8%)
			Stone formation	2/68 (2.9%)
